# Multidrug Resistance and Cancer Stem Cells in Neuroblastoma and Hepatoblastoma

**DOI:** 10.3390/ijms141224706

**Published:** 2013-12-18

**Authors:** Anna Alisi, William C. Cho, Franco Locatelli, Doriana Fruci

**Affiliations:** 1 Liver Research Unit, “Bambino Gesù” Children’s Hospital, IRCCS, Rome 00165, Italy; 2 Department of Clinical Oncology, Queen Elizabeth Hospital, 30 Gascoigne Road, Kowloon, Hong Kong, China; E-Mail: williamcscho@gmail.com; 3 Department of Oncohematology, “Bambino Gesù” Children’s Hospital, IRCCS, Rome 00165, Italy; E-Mail: franco.locatelli@opbg.net

**Keywords:** cancer stem cells, multidrug resistance, neuroblastoma, hepatoblastoma

## Abstract

Chemotherapy is one of the major modalities in treating cancers. However, its effectiveness is limited by the acquisition of multidrug resistance (MDR). Several mechanisms could explain the up-regulation of MDR genes/proteins in cancer after chemotherapy. It is known that cancer stem cells (CSCs) play a role as master regulators. Therefore, understanding the mechanisms that regulate some traits of CSCs may help design efficient strategies to overcome chemoresistance. Different CSC phenotypes have been identified, including those found in some pediatric malignancies. As solid tumors in children significantly differ from those observed in adults, this review aims at providing an overview of the mechanistic relationship between MDR and CSCs in common solid tumors, and, in particular, focuses on clinical as well as experimental evidence of the relations between CSCs and MDR in neuroblastoma and hepatoblastoma. Finally, some novel approaches, such as concomitant targeting of multiple key transcription factors governing the stemness of CSCs, as well as nanoparticle-based approaches will also be briefly addressed.

## 1. Introduction

Chemotherapy is the treatment of choice against a variety of cancers. Its effectiveness is, however, limited by the acquisition of multidrug resistance (MDR) of cancer cells that become insensitive not only to the primary cytostatic drug used, but also to other pharmaceutical agents structurally distant to the cytostatic drugs [1].

Cancer cells may either exhibit a significant primary resistance to chemotherapeutic drugs (intrinsic resistance), or acquire characteristics of MDR during chemotherapy (acquired resistance). Three major mechanisms have been proposed to explain MDR in cancer: first, decreased uptake of water-soluble drugs, such as cisplatin, which requires transporters to enter cells; second, changes in cancer cells that affect their susceptibility to cytotoxic drugs, including alteration of fundamental cellular processes, such as cell cycle control, apoptosis, DNA repair and metabolism of drugs; third, increased energy-dependent efflux of hydrophobic cytotoxic drugs that enter cells by diffusion through the plasma membrane keeping intracellular concentrations below the cell-killing threshold [1–5]. The efflux of cellular cytotoxic drugs, which is the mechanism most commonly investigated, is attributed to the overexpression of a family of energy-dependent multi-drug transporters known as ATP-binding cassette (ABC) transporter proteins. These trans-membrane proteins use the energy from the hydrolysis of ATP to actively export drugs from the cell thus avoiding the drug-related toxic effects [1–5].

Malignant tumors are heterogeneous diseases characterized by a broad range of morphological and functional phenotypes. This heterogeneity can be explained either by the classical stochastic model based on somatic mutations occurring in all tumor cells within tumor bulk, or by a hierarchical model according to which only a small subpopulation of cells within tumor bulk leads to tumor initiation, growth, recurrence and metastasis [6,7]. These cells, called “cancer stem cells” (CSCs), exhibit characteristics similar to normal stem cells, including self-renewal, differentiation capacity and tumorigenicity in xenotransplants [8,9].

Nowadays, we know that CSCs may play a role as master regulators not only in the development of several solid tumors but also during the process of chemoresistance acquisition [10,11]. Therefore, the comprehension of the mechanisms that regulate some traits of CSCs may help design efficient strategies to overcome chemoresistance [12,13]. Different CSC phenotypes have been identified in several adult-type solid tumors [14–16] and, more recently, in some pediatric malignancies [17]. Given the significant difference in the biology of pediatric and adult tumors, this, in particular, being due to their different origins, it is of interest to explore the relevance of CSCs in MDR and their potential role in response to treatment in childhood neoplasia.

This review provides an overview of the current knowledge of the mechanistic relationship between MDR and CSCs in two common pediatric solid tumors, neuroblastoma and hepatoblastoma, focusing in particular, on the clinical and experimental evidence of the relationship existing between CSCs and MDR.

## 2. MDR in Neuroblastoma and Hepatoblastoma

### 2.1. MDR Concepts and Proteins

The *ABC* genes represent the largest family of trans-membrane proteins and generally use energy derived from ATP hydrolysis to drive the transport of different substrates (drugs, drug metabolites, and endogenous metabolites) across biological membranes, a process critical for most aspects of cell biology.

Members of the ABC family are classified according to the sequence and organization of their ATP-binding domain(s) also known as nucleotide-binding domains (NBD) [18]. In addition to the Walker A and Walker B motifs that are typical of all ATP-binding proteins, the ATP-binding domains of ABC transporters contain the so-called “ALSGGQ” signature motif or “C” motif, which has been directly implicated in the recognition, binding, and hydrolysis of ATP. The functional ABC transporters typically contain two nucleotide-binding folds and two *trans*-membrane (TM) domains, expressed either as one unit or in two separate units. TM domains may contain from 6 to 12 membrane-spanning alpha helices, which provide the specificity for the substrate. NBDs are located in the cytoplasm and are supposed to transfer the energy needed to transport the substrate across the membrane.

Most of the known functions of eukaryotes ABC pumps predominantly involve the export of hydrophobic compounds from the cytoplasm to the outside of the cell or into an extracellular compartment (endoplasmic reticulum, mitochondria, peroxisome).

In humans, 49 genes encode for ABC family members belonging to seven different subfamilies designed A through G [18,19]. The normal function of human ABC transporters is to detoxify and protect the body from cytotoxic compounds, such as endogenous cytotoxic metabolites and drugs. Tissue distribution of ABC transporters through pharmacological barriers, such as the brush border membrane of intestinal cells, the biliary canalicular membrane of hepatocytes, the luminal membrane of proximal tubules of the kidney, and in the epithelium of the blood-brain barrier, reflects their function. Loss-of-function mutations in some of the ABC genes results in generation of genetic disorders, such as cystic fibrosis, anemia, a bleeding disorder called Scott syndrome, and a number of eye and liver diseases [20–22].

**Figure 1.**
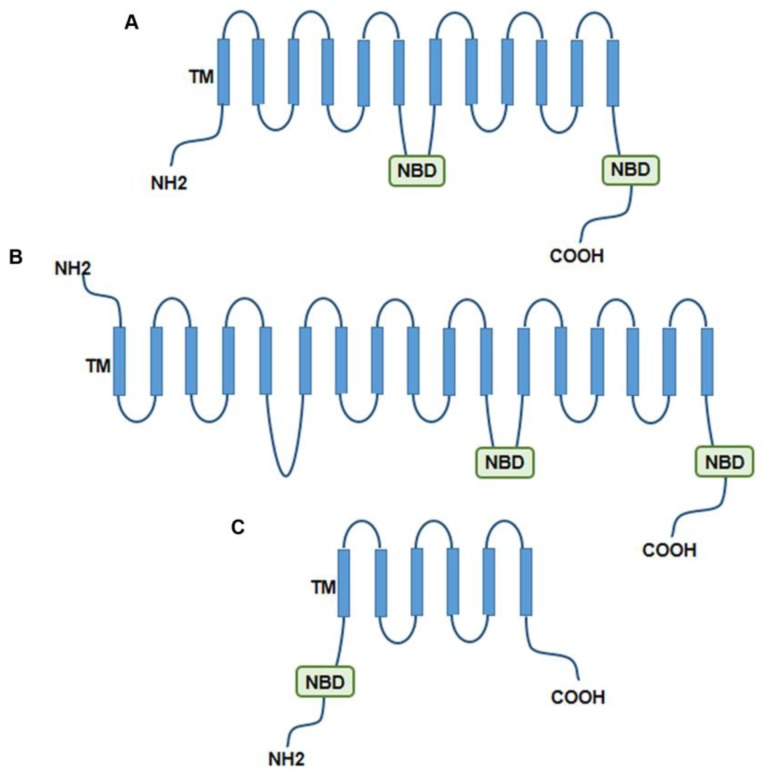
Structure of the ABC transporters found to confer multidrug resistance (MDR) in cancer. (**A**) Glycoprotein P (P-gp); (**B**) Multidrug resistance-associated protein 1 (MRP1); (**C**) Human breast cancer resistance protein (BCRP).

The three pumps commonly found to confer MDR in cancers are the ABC protein glycoprotein P (P-gp), the so-called multidrug resistance-associated protein 1 (MRP1) and the human breast cancer resistance protein (BCRP). Because of their function and importance, they are the targets of several anticancer investigations. The structure of these ABC transporters is shown in Figure 1.

P-gp is a 170 kDa glycoprotein encoded by the ABCB1 gene (also termed multidrug resistance protein 1 (MDR1) gene) [23], which regulates the export of approximately 20 structurally unrelated anticancer agents from the cell, including paclitaxel, doxorubicin, and vincristine. This protein is normally expressed in tissues that are strategically located to protect against the passage of xenobiotics, including the bronchopulmonary epithelium, hepatobiliary epithelium, renal tubular epithelium, gastrointestinal tract, blood-brain barrier and choroid plexus. Its differential expression in cells of the hematopoietic system, including antigen-presenting cells, subpopulations of T and B lymphocytes and natural killer cells, implies diverse physiologic and pharmacologic roles [24]. As expected, P-gp expression is the highest in tumors derived from tissues that normally express P-gp. However, in many other tumors, the expression of P-gp is induced by chemotherapy.

MRP1 (also known as ABCC1) is a 190 kDa protein widely expressed in normal tissues with relatively higher levels in lung, testis, kidney, and peripheral blood mononuclear cells. This transporter has been found to be up-regulated in a variety of solid tumors, including those of lung, breast, and prostate [25]. MRP1 expression constitutes a negative prognostic marker for early-stage breast cancer [26–29] and is predictive of poor response to chemotherapy and dismal survival in non-small cell lung carcinoma (NSCLC) and small cell lung carcinoma (SCLC) [30–34]. Its substrate specificity is broadly similar to that of P-gp, from which it diversifies for the export of organic anions, e.g. drugs conjugated to glutathione (GSH), glucuronate, or sulphate [35].

BCRP, also known as ABCG2, took its name from the multidrug resistant breast cancer cell line co-selected for doxorubicin and verapamil resistance from which it was isolated. BCRP is a small protein (70 kDa) consisting of a single NBD and MDS, which is thought to dimerize with another protein to form a functional transporter. It is capable of transporting doxorubicin, mitoxantrone, topotecan, methotrexate, and tyrosine kinase inhibitors, among other substances. This transporter is expressed in a variety of normal tissues with the highest levels found in the placenta, as consistent with the hypothesis of a protective role for the fetus. BCRP protein has been found overexpressed in several multi-drug resistant tumors.

Increased expression of MDR-associated genes has been reported in various primary untreated pediatric tumors, including neuroblastoma and hepatoblastoma [36,37]. These results are obtained by evaluating expression of MDR genes at mRNA and protein levels by quantitative PCR and immunohistochemistry. Of note, Oue *et al*. have confirmed through immunohistochemical analysis that most of these proteins present a specific pattern of expression in several post-chemotherapy pediatric solid malignancies [38].

### 2.2. Neuroblastoma and MDR

#### 2.2.1. Epidemiology and Characteristics of Neuroblastoma

Neuroblastoma (NB) is a childhood solid tumor that originates from progenitor cells of the sympathetic nervous system and represents the third leading cause of cancer-related death in children [39,40]. It has a heterogeneous clinical behavior, ranging from spontaneous regression to rapid progression, which is associated with several biological and genetic factors, including age at diagnosis, tumor stage, histology and the presence of genetic and chromosomal abnormalities. The most widely characterized genetic changes associated with a worse outcome are amplification of the *MYCN* oncogenic transcription factor, ploidy, deletion or loss of heterozygosity of loci on 1p36 or 11q23, and unbalanced gain of distal 17q [39,40].

Despite aggressive treatment, including high dosage of chemotherapy followed by myeloablative cytotoxic therapy with autologous hematopoietic stem cell transplantation, local radiotherapy and differentiation therapy with 13-*cis-*retinoic acid, the clinical outcome of these patients (accounting for 50% of all NBs) still remains poor, with less than 40% long-term remission [39,40]. One of the major factors that contribute to the chemotherapeutic treatment failure of high-risk NB patients is the acquisition of drug resistance by tumor cells. *MYCN* clearly contributes to this phenomenon in NB, as amplification of this transcription factor is strongly associated with rapid progression and poor prognosis [41,42].

#### 2.2.2. Clinical/Experimental Evidence of MDR in NB

The first evidence of an association between high levels of MRP1 expression in primary tumors and amplification and overexpression of MYCN dates back to 1994 [43]. The authors also found that the treatment of NB cell lines with retinoic acid resulted in coordinated down-regulation of *MYCN* and *MRP1*. Since then, the coordinated expression between these two genes has been confirmed in a larger number of tissue samples and NB cell lines [44–49]. In a retrospective analysis of 60 primary NB specimens, increased levels of MRP1 expression were strongly associated with reduced overall survival and event-free survival and they were found to predict outcome also in patients with MYCN non-amplified tumors [44]. Further evidences of a role for MRP1 in NB have been provided by another study that examined expression of MRP1 and P-gp in a cohort of 70 primary untreated tumors [50]. Of these two proteins, only MRP1 was significantly associated with patient outcome [50]. P-gp, in fact, appears to have a more limited role in the development of drug resistance in NB [51–55].

Several authors have also provided clear evidences that MRP1 is a direct transcriptional target of *MYCN* in NBs [45,56]. Specifically, MYCN regulates the *MRP1* gene expression through interaction with *cis*-acting factors in the MRP1 promoter. MYCN induction resulted in increased MPR1 expression, which in turn was followed by increased drug resistance and enhanced MRP1-mediated drug efflux [56], suggesting that *MRP1* may be a MYCN target gene involved in the MDR phenotype of NB. More recently, Henderson *et al.* demonstrated that MRP1 contributes to the development of NB in a mouse model [57] and that, in addition to MRP1, other MRP family members contribute to drug resistance in NB. Specifically, an increased expression of MRP4 (ABCC4) was strongly predictive of poor clinical outcome in the most aggressive forms of NB. The authors also found a correlation between expression of MRP4 and MYCN [58], reflecting a possible common regulatory mechanism. To confirm this result, a recent study demonstrated that MYCN positively regulates the expression of both transporters, MRP1 and MRP4 [59]. These findings suggest that MRP1 and MRP4 may facilitate development of aggressive and therapy-resistant forms of NB.

### 2.3. Hepatoblastoma and MDR

#### 2.3.1. Epidemiology and Characteristics of Hepatoblastoma

Hepatoblastoma (HB) is the most frequent pediatric liver cancer accounting for 79% of all primary malignant hepatic tumors in children before age 3 years, with a worldwide incidence rate that is difficult to evaluate due to important differences across various ethnicities [60].

In a recent consensus statement, the cooperative Children’s Oncology Group (COG) histologically classified HB in two major types: epithelial and mixed types accounting for 56% and 44% of the cases, respectively [61]. As HB arises from hepatocellular precursors, it is characterized by a combination of different phenotypes that resemble the patterns displayed during liver development. In fact, epithelial and mixed epithelial/mesenchymal types can present several cellular and tissue morphologies. The epithelial type includes pure fetal, embryonal, macrotrabecular, small-cell undifferentiated and cholangioblastic subtypes, while mixed type may also contain stromal derivatives (*i.e.*, blastema, osteoid, skeletal muscle and cartilage) or heterologous components, such as endoderm, neuroectodermal derivates, and melanin-containing cells, in the teratoid variant. Liver biopsy is crucial for diagnosis and to design an appropriate interventional strategy, because it permits to stage HB by evaluating the amount of liver mass involvement and eventually the intra- and extra-hepatic tumor spreading [61]. Although surgical resection is the keystone of treatment for patients with HB, its efficacy strongly depends on the exact characterization of the tumor. In fact, in some cases, neoadjuvant pre-surgical chemotherapy is recommended before definitive resection in order to obtain a reduction of tumor size and/or a clear distinction between the tumor mass and the surrounding liver tissue [62]. Currently, the International Society of Pediatric Oncology Epithelial Liver Tumor Group (SIOPEL) utilizes a pre-surgical-based staging system termed PRETEXT, while COG employs a postsurgical-based staging system and is now evaluating the usefulness of PRETEXT combined with a POSTTEXT staging system after chemotherapy to determine the optimal treatment strategy [61–63].

#### 2.3.2. Clinical/Experimental Evidence of MDR in HB

Most cases of HB display a good response to pre-surgical neoadjuvant chemotherapy, while some others, particularly multifocal non-metastatic HB, remain unresectable after chemotherapy due to the high risk of leaving viable malignant tumor cells. In these latter cases, the only effective treatment is represented by liver transplantation, which has serious limitations including the scarcity of donors, the risk of surgical complications, possible organ rejection, and high cost [64]. Therefore, alternative strategies aimed at enhancing the response to pre-surgical chemotherapy, as well as innovative cell-based liver-directed therapies, are desirable.

MDR is considered one of the major reasons for poor response to chemotherapy in high-risk and recurrent HB [37]. Warmmann *et al.* demonstrated that, in a child with multifocal HB, *MDR1* gene expression was strongly up-regulated (approximately six times) during the clinical course from pre-surgery to the second cycle of post-surgery chemotherapy. These results are in agreement with a more recent study demonstrating that, while P-gp and MRP1 expression is up-regulated, LRP is neo-expressed in HB after chemotherapy [37,38].

Research studies on the existence and role of MDR in HB frequently used several available *in vitro* continuous cell lines and *in vivo* animal models demonstrating up-regulation of P-gp after standard chemotherapy with cisplatin and doxorubicin [37,65,66].

Interestingly, *in vitro* and *in vivo* modulation of MDR genes/proteins improves the response of HB to chemotherapy [67,68].

Two possible mechanisms could explain the up-regulation of MDR genes/proteins in HB after chemotherapy: the first mechanism hypothesizes a regulatory network potentially associated with the expression of other genes/proteins, while the second assumes an event of clonal selection. Therefore, it is conceivable that different histological subtypes of HB could display a diverse expression and response of MDR genes/proteins to chemotherapy. It can be expected that MDR in HB, like in other pediatric solid malignancies could be associated with presence either of well-differentiated tumor cells and/or of specific immature cells (like CSCs) [69]. The experimental 3D cell-culture model recently developed by Eicher *et al.* could be a good approach to investigate MDR in different cell sub-populations isolated from primary HB [70].

## 3. CSCs in NB and HB

### 3.1. General Concepts and Characteristics of CSCs

The multistep stochastic model for cancer development is based on the nature and number of successive mutations, while the CSC theory proposes that highly defined populations of cells retain the capacity to promote tumor growing and spreading [6,7]. Despite the ongoing intense debate on the existence, the potential origin and functional characteristics of CSCs, there is large agreement on the fact that these cells are heterogeneous in most cancers and can constantly change during disease progression. However, a tumor could start in a way that follows the stem cell developmental hierarchy and further progress with the acquisition of mutations in a way that resembles the clonal evolution model [13]. Thus, the CSC theory assumes a hierarchical structure in a tumor in which it is not only considered as the *primum movens* of cancer, but also as the real engine of its progression.

Hence, to understand the potential role and evolution of CSCs we need a clear definition of CSCs and information about recognition patterns capable of discriminating them from other cells in different malignancies.

The American Association for Cancer Research (AACR) in 2006 provided a consensus definition for CSCs suggesting the minimum descriptors that allow identifying them: “the capacity to self-renew and to cause the heterogeneous lineages of cancer cells that comprise the tumor” [71]. However, CSCs are currently defined by four major traits: self-renewing capacity; differentiation capacity; tumor-initiating capacity, and metastatic potential [13].

**Figure 2.**
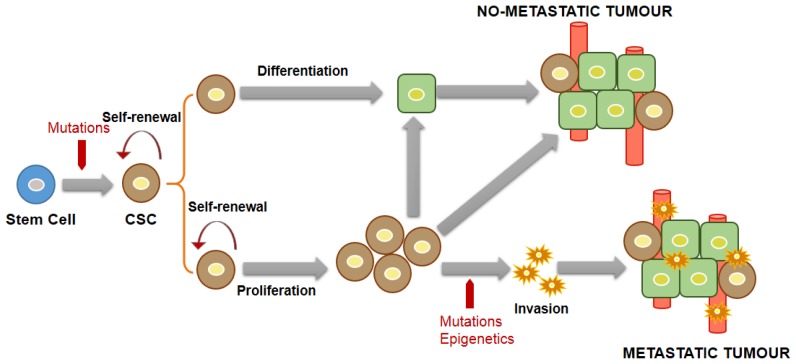
Representative model of cancer stem cells (CSCs)-driven tumorigenesis.

These defining requirements can be justified by a combination of CSC theory and a clonal evolution model. According to this mixed model (Figure 2), during tumor development and progression, a CSC, originating from a stem cell under the pressure of mutagenic hits, self-renews by symmetric and asymmetric division and generates, on the one-hand, tumor-initiating cells (TICs) able to differentiate and cause tumor formation, and, on the other hand, a progeny of CSCs which retain self-renewing capacity, while maintaining their pluripotent state and increasing the tumor mass. This last pluripotent subset of CSCs with its huge proliferative ability could sustain tumor progression and be subjected to mutation or epigenetic modifications that are responsible for its invasive properties leading to metastasis.

Operationally, specific cell-surface marker profiles have been identified in order to characterize and investigate both the behavior of CSC subpopulations and the way in which they recapitulate the cellular heterogeneity of the tumors [72]. Although the CSC marker profile is usually similar in tumors of the same type, three major markers are expressed across different tissue types: CD133, which stains proliferative cells in multiple organs, CD44, which is broadly expressed in many tumors, and the epithelial cell adhesion molecule EpCAM, which is a pan-epithelial marker [13].

As the different phenotypes of CSCs can possibly derive from both embryonic and adult stem cell pools, as well as from progenitor and differentiated cells, several markers have been described in solid tumors, including breast, skin, brain, colon, and liver cancers [73,74]. A CD133 positive CSC subpopulation has been identified in two cancers frequently diagnosed in children, such as osteosarcoma and rhabdomyosarcoma [75]. CD133 positive CSCs with self-renewal properties were isolated and identified in osteosarcoma cell lines and in two osteosarcoma tissues, even if the tumorigenicity of these cells was not evaluated through *in vivo* xenotransplant assays [76,77]. Walter *et al*. demonstrated that high expression of CD133 in rhabdomyosarcoma correlated with survival rate of patients and that the CD133 positive cells isolated from rhabdomyosarcoma cell lines generated tumors in NOD/SCID mice [78].

In addition to the above-mentioned markers, two other characteristics of CSCs have been exploited for their identification and isolation: the side-population analysis and the anchorage-independent sphere formation ability. Side population cells were firstly described during the isolation of hematopoietic stem cells and then reported in many tumors [71,79]. With the term of side population is defined a subset of CSCs that expresses ABC transporters and that can be identified by their ability to rapidly efflux the Hoechst 33342 DNA-binding dye [80]. Although studies indicated that the percentage of side population cells change among different tumors, some evidences suggest that data on side population may be used as prognostic indicator and as test to evaluate efficacy of chemotherapy [81,82].

The anchorage-independent sphere formation assay was instrumental in the study of adult stem cells including nerve, prostate, and mammary stem cells [83,84]. Recently, as CSCs have also been identified based on their ability to form colonies *in vitro*, this approach has been used to enrich the potential CSC subpopulations when their specific markers were unknown [85,86]. In this case, isolated cells can be cultured in serum-free media containing epidermal growth factor and basic fibroblast growth factor, and the development of spherical colonies is considered indicative of self-renewal ability and of CSC phenotype.

Despite the fascinating properties of CSCs, the role of these cells in tumorigenesis, as proposed by CSC theory, is not written in stone and it is still a motif of intense scientific debate. Therefore, the consistency of CSC theory in different tumor backgrounds requires extreme caution.

### 3.2. CSC Markers in NB

A subpopulation of stem cells has been identified in certain NB cell lines (I-type NB cell lines) having a high capacity to form colony and grow in immunodeficient mice [87]. The percentage of CSCs, defined by expression of the CD133 marker, was more abundant in NB than in ganglioneuroblastoma and found to correlate with the clinical stage, being higher in tumors with unfavorable rather than in those with favorable histology. The authors found that patients with CD133-positive tumor cells have an overall survival shorter than those with CD133-negative tumor cells [88].

Knockdown of CD133 inhibits differentiation of NB cell lines and primary tumor cells [89], as well as sphere formation, suggesting a role of CD133 in NB cell stemness [90]. As compared to cells cultured in medium in the presence of serum, the cell forming sphere isolated from highly malignant NB samples is resistant to doxorubicin, cisplatin, and etoposide. Hansford *et al*. studied the tumor-initiating capacity of cells isolated from bone marrow metastasis of NB patients [91]. They found that these cells formed metastases and grew as spheres in a serum-free medium, contained chromosomal aberrations typical of NB and were self-renewing [91]. Cournoyer *et al*. found that CD133-positive cells isolated from six NB cell lines have gains on 16p13.3, 19p13.3, and 19q13.33 that are on the contrary absent in CD133-negative cells. The authors also found a correlation between the presence of a gain of 16p13 and expression of CD133 in 26 samples of NB [92].

Similar to other tumors, NB CSCs are sensitive to telomerase inhibition. Because normal tissue stem cells lack telomerase activity, telomerase inhibition resulted in CSC exhaustion by irreversibly altering their self-renewal capacity [93]. Some authors identified two compounds (DECA-14 and rapamycin) that selectively targeted NB CSC, while having little effect on normal stem cells, preventing NB CSC self-renewal both *in vitro* and *in vivo*.

CD133-positive cells from different chemoresistant cancers are enriched *in vivo* after treatment with cisplatin, etoposide, doxorubicin, and paclitaxel [94,95]. The NB side population cells expressed high levels of BCRP and ABCA3 transporter genes and had capacity to expel mitoxantrone, resulting in better survival of side population cells cultivated with this drug [96]. BCRP transports also anthracyclines, imatinib, and topoisomerase I and II inhibitors. The CD133-positive cells isolated from NB cell lines are more resistant to cisplatin, carboplatin, etoposide, and doxorubicin than the CD133-negative ones [97].

### 3.3. CSC Markers in HB

Although HB is a malignant embryonal tumor of the liver, characterized by a distinct morphological pattern reminiscent of hepatoblasts, only few studies reported the presence of CSC traits in this tumor [98–102]. Expression of EpCAM was found in all tumor-resident epithelial cells of 70%–80% of cases of HB [98,99]. Accordingly, immunohistochemical analysis of 61 hepatoblastomas reported that EpCAM expression was found in 83.6% of cases [100]. More, recently, Armeanu-Ebinge *et al*. investigated EpCAM as a target for immunotherapy in two HB cell lines, HUH6 and HepT1 [101]. In this study, the authors found that EpCAM was constantly expressed on cultured HB cell lines, independently of cisplatin-based chemotherapeutic treatment and demonstrated that exposure of γδ T cells to EpCAM-specific monoclonal antibodies strongly increased lysis and reduced viability of cancer cells.

Akita *et al*. demonstrated by atmospheric scanning electron microscope that HUH6 cells exhibited CD133 positivity preferentially in membrane ruffles [102]. Next, electron microscopy revealed that CD133 protein was associated to a complex structure comprising filopodia and the leading edge of lamellipodia and co-localized with F-actin. Furthermore, as an antibody against CD133 decreased migration of HUH6 cells, the authors suggested that this protein could play a role in tumor invasion and metastasis. Interestingly, also Hep293TT, derived from a 5-year-old child with an aggressive HB with features of transitional cell liver tumor, expressed CD133, the positivity being reduced by treatment with bortezomib and sorafenib [103].

Additional histochemical analyses demonstrated that HB exhibits different types of stem cells that could play a specific role during CSC-dependent tumorigenesis [104,105]. The expression of these stem cell markers and molecular signatures divide HB in two classes: poorly-differentiated types, which express fewer stem cell markers, and well-differentiated types that contrarily exhibit positivity for most of these proteins and are less aggressive [104]. Lingala *et al*. found that only HB encapsulated by connective tissue displayed CD90, CD44 and CD133 positivity, while the liver tissue surrounding the neoplasm was completely negative for all of these proteins [105].

Immunohistochemical positivity to OV6 and OV1 antibodies, which recognize antigens associated with hepatic stem oval cells, was also found in HB [106]. However, since other authors reported the absence of an oval cell phenotype in HB, the role of these CSC markers in the histogenesis of HB is still unclear [107]. OV6-positive cells showed a characteristic co-expression with other CSC markers including CD90 and CD34 in hepatocellular carcinoma [108]. Interestingly, Fiegel *et al.* demonstrated by immunohistochemical analysis that these two markers were also expressed in tissue samples from HB [109].

The possible existence of CSCs in HB was also confirmed by two *in vivo* studies on mice [110,111]. The first study demonstrated that single cell cultures of Huh6 cells were able to generate spontaneously germ cell-like cells and embryo-like derivatives that induce tumor formation in xenotransplant [110]. The second study demonstrated that a side population from a human HB cell line was able to form tumors in mice whereas tumor formation was not observed in the non-side population cells [111].

Finally, a recent study demonstrated the existence of oncogene-specific formation of chemoresistant murine hepatic cancer stem cells [112]. In this study, the authors found an increased expression of MDR1 in the enriched side population CSCs that was able to explain in a mouse model of HB their functional chemoresistance against paclitaxel and doxorubicin.

## 4. Association between CSCs and MDR and Its Diagnostic Significance

The development of drug resistance remains a fundamental problem to be overcome in the cure of cancer. In fact, current chemotherapeutic agents are effective against the CSCs, but in several cancers a residual pool of this subset of cells remains, determining recurrence of disease [69,113]. CSCs that survive after chemotherapy gave rise to a population of chemoresistant cells able to sustain the growth of a more aggressive and potentially metastatic tumor. Therefore, as defined by Cordon-Cardo, chemoresistant CSCs could be considered the Achilles’ heel of cancer [114].

Several mechanisms could explain the genesis of CSC chemoresistance including resistance to DNA damage and apoptosis, and the ability to make epithelial to mesenchymal transition [10]. However, usually, cancers that recur after an initial response to chemotherapeutic agents become resistant to these and other drugs, because of the phenomenon of MDR. In fact, among the several protective mechanisms for CSCs, ABC protein overexpression is probably the most important one.

**Figure 3.**
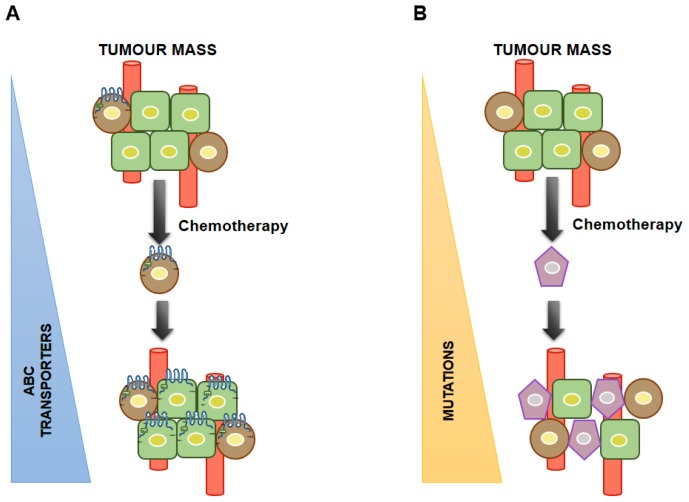
Models to explain drug resistance in CSCs. (**A**) Original drug resistance model; (**B**) Acquired drug resistance model.

Two models, have been proposed to explain the origin of CSC MDR in tumors with an elevated ability to survive conventional chemotherapeutic regimens (Figure 3). The first model proposes that after exposure to the chemotherapeutic agent, only the CSCs expressing ABC transporters are able to repopulate the tumor by asymmetrical cell division with newly-formed CSCs and/or differentiated progenitor cells. The second acquired model suggests that after chemotherapy, only CSCs survive and, those that acquire drug resistance under the pressure of mutations, originate new and more aggressive drug-resistant cell phenotypes.

A further complication of the complex interactions between CSCs and MDR is represented by the pressure of the niche microenvironments that, as in the case of the stochastic model of cancer, may play a major role in determining maintenance, flexibility, and chemoresistant properties of these cells [115].

Although there is evidence for both models, more research effort is required to confirm CSC theory and to fully appreciate the complexity of mechanisms regulating properties and functions of CSCs, and particularly to crack codes to decipher their links to MDR in pediatric tumors.

## 5. Future Perspective and Conclusions

Although several questions remain open about CSC theory, there is mounting evidence that chemoresistant CSCs can be considered the Achilles’ heel of cancer. Among the several protective mechanisms for CSCs, ABC protein overexpression is probably the most important.

Currently, there are ongoing efforts to exploit the efficacy of concomitant targeting of multiple key transcription factors governing the stemness of CSCs in suppressing CSC-like phenotypes. Targeting the key genes conferring stemness to CSCs can efficiently eliminate CSC-like phenotypes, and thus may be considered to provide a new approach to cancer therapy [106]. It is foreseeable that the combination of multiple targeting as a potential anti-cancer agent will be further studied in this direction.

Moreover, nanoparticle-based approaches have been investigated to overcome efflux-mediated resistance, including the formulation of excipients that inhibit transporter activity and co-delivery of an anticancer drug with a specific inhibitor of transporter function or expression. There is ongoing improvement in the intra-tumoral distribution of nanoparticles by adjunct therapies, which may be vital to the successful application of nanotechnology to overcome tumor drug resistance [107].
